# Spatial Analysis of Drug-Susceptible and Multidrug-Resistant Cases of Tuberculosis, Ho Chi Minh City, Vietnam, 2020–2023

**DOI:** 10.3201/eid3003.231309

**Published:** 2024-03

**Authors:** Ruan Spies, Hanh N. Hong, Phu P. Trieu, Luong K. Lan, Kim Lan, N.N. Hue, Nguyen T.L. Huong, Tran T.L.N. Thao, Nguyen L. Quang, Thu D.D. Anh, Truong V. Vinh, Dang T.M. Ha, Phan T. Dat, Nguyen P. Hai, Le H. Van, Guy E. Thwaites, Nguyen T.T. Thuong, James A. Watson, Timothy M. Walker

**Affiliations:** University of Oxford, Oxford, UK (R. Spies, G.E. Thwaites, N.T.T. Thuong, J.A. Watson, T.M. Walker);; Oxford University Clinical Research Unit, Ho Chi Minh City, Vietnam (H.N. Hong, P.P. Trieu, L.K. Lan, K. Lan, N.N. Hue, N.T.L. Huong, T.L.T.N. Thao, N.L. Quang, T.D.D. Anh, L.H. Van, G.E. Thwaites, N.T.T. Thuong, J.A. Watson, T.M. Walker);; Pham Ngoc Thach Hospital, Ho Chi Minh City (T.V. Vinh, D.T.M. Ha, P.T. Dat, N.P. Hai)

**Keywords:** tuberculosis, TB, tuberculosis and other mycobacteria, bacteria, respiratory infections, spatial analysis, spatial heterogeneity, drug-susceptible TB, multidrug-resistant TB, Ho Chi Minh City, Vietnam

## Abstract

We characterized the spatial distribution of drug-susceptible (DS) and multidrug-resistant (MDR) tuberculosis (TB) cases in Ho Chi Minh City, Vietnam, a major metropolis in southeastern Asia, and explored demographic and socioeconomic factors associated with local TB burden. Hot spots of DS and MDR TB incidence were observed in the central parts of Ho Chi Minh City, and substantial heterogeneity was observed across wards. Positive spatial autocorrelation was observed for both DS TB and MDR TB. Ward-level TB incidence was associated with HIV prevalence and the male proportion of the population. No ward-level demographic and socioeconomic indicators were associated with MDR TB case count relative to total TB case count. Our findings might inform spatially targeted TB control strategies and provide insights for generating hypotheses about the nature of the relationship between DS and MDR TB in Ho Chi Minh City and the wider southeastern region of Asia.

Tuberculosis (TB) causes more deaths worldwide than any other infectious disease. Progress in reducing the global burden of TB stalled during the COVID-19 pandemic; an estimated 10.6 million persons became ill from TB in 2021, and 1.6 million died (*1*). The number of persons with multidrug-resistant TB (MDR TB), defined by resistance to rifampin and isoniazid, is estimated to have increased by 3.1% since 2020 (*1*), including an estimated 450,000 incident cases in 2021. MDR TB remains underdiagnosed and is associated with worse treatment outcomes than for drug-susceptible TB (DS TB) (*1*,*2*).

TB is spatially heterogeneous both globally and locally. Thirty low- and middle-income countries account for nearly 90% of the global burden of disease (*1*), but an unequal distribution of disease has also been described more locally (*3*–*12*). Although poorly understood, the drivers of geographic heterogeneity in TB are believed to reflect the complex interplay between the infectious and susceptible host, the infecting organism, the physical environment, and distal determinants such as poverty (*13*).

The World Health Organization (WHO) recognizes Vietnam as a high-burden country for TB and MDR TB; estimated incidence is 173 (95% CI 112–247) cases/100,000 population for TB and 9.1 (95% CI 5.5–13) cases/100,000 population for MDR TB (*1*,*14*). The highest incidence is seen in the southern parts of the country, especially in Ho Chi Minh City (*15*,*16*). Patients with MDR TB in Ho Chi Minh City can have acquired their disease through selection of drug-resistance mutations while receiving first-line TB drug treatment or directly from others through transmission (*17*). Comparison of the spatial distributions of DS and MDR TB across this high-incidence city has the potential to offer insights into relative contributions of each to MDR TB burden. For example, the observation of distinct spatial distributions of DS and MDR TB might support the hypothesis that MDR TB is transmitted in networks independent from circulating DS TB. Alternatively, sporadic MDR TB cases among clusters of DS TB cases might be more indicative of de novo emergence of MDR TB through inadequate treatment and selection. Clarifying hyperlocal patterns of disease might also contribute to spatially targeted interventions, such as active case finding and healthcare facility planning (*18*–*21*), and to the design of and recruitment into clinical trials and other studies. In this study, we aimed to characterize the spatial distribution of DS and MDR TB in Ho Chi Minh City and to explore demographic and socioeconomic factors associated with local TB burden. 

## Methods

### Study Setting

Ho Chi Minh City has a total population of ≈10 million persons and is subdivided into 24 districts, 19 urban and 5 rural (Appendix Figure 1), of which 3 were combined to form a municipal city, Thủ Đức City, in 2021. Districts of Ho Chi Minh City are further subdivided into 322 administrative subunits consisting of wards, townlets, and communes (hereafter wards); median population is ≈22,000 persons. This study includes data from before 2021 and therefore references the previous 24-district subdivision of Ho Chi Minh City.

Public-sector community-based TB care in Ho Chi Minh City is coordinated through 24 district treatment units (DTUs), where persons with suspected TB are referred for testing and treatment. Once given a diagnosis of TB, patients are registered with the National TB Program (NTP). All persons given a diagnosis of MDR TB in the public sector initiate treatment through the city’s lung hospital, Phạm Ngọc Thạch, and then continue outpatient care through the DTUs. Phạm Ngọc Thạch Hospital is the regional center for MDR TB treatment in southern Vietnam and provides treatment for ≈80% of all MDR TB cases in Vietnam (*22*).

### Study Population

The study population included all persons who registered for TB treatment in the public sector in 23 of the districts of Ho Chi Minh City during January 1, 2020–April 30, 2023. The study excluded TB cases from Cần Giờ, a rural district comprising 7 wards with a population of 71,527 persons (0.8% of the population Ho Chi Minh City) (*23*), because data were not available. For the ecologic analysis, 315 residential wards constituting 23 of the districts of Ho Chi Minh City formed the units of analysis.

### Data Sources

We accessed data for participants with DS TB from the Vietnam TB Information Management Electronic System, a web-based surveillance system that records TB notifications and treatment outcomes for the NTP (*24*). This system includes data on all persons in Ho Chi Minh City initiated on first-line TB therapy in the public sector. At treatment initiation, patient details are added to a paper-based register, which is electronically transcribed by DTU staff at monthly intervals. Data extracted from the electronic register for this study included participant age, sex, home address, HIV status, and history of previous TB. We obtained data for participants with MDR TB from an ongoing cohort study conducted through the Oxford University Clinical Research Unit. Participants included all persons initiating treatment for MDR TB at Phạm Ngọc Thạch Hospital. We selected the Oxford University Clinical Research Unit cohort study database as the data source for MDR TB cases because it provided identical case coverage to the NTP-based register, with less missing data.

We obtained district-level and ward-level demographic and socioeconomic indicators from published regional data collected as part of the 2019 Vietnam census (*23*). Extracted indicators that were available at only the district level were population age structure, unemployment rate, proportion of households that had a computer, and number of persons living with HIV. All wards within a district were assigned the district value for indicators available only at the district level. For example, District 1 had an HIV prevalence of 1.5%; this value was subsequently assigned to each of the constituent wards of District 1. Extracted indicators, which were available at the ward level, were total population, population by sex, population density, average number of persons per household, literacy rate, and residence type (urban or rural). Location was labeled as city center if wards were located in the central commercial, commuting, and socializing hubs of Ho Chi Minh City and as peripheral if wards were located outside those areas (Appendix).

### Design and Analysis

We used individual-level data for a descriptive, cross-sectional analysis of the burden of TB in Ho Chi Minh City and the characteristics of TB cases. We used an ecologic design, using ward-level data, to describe ward-level factors associated with TB burden. The outcomes for the ecologic analysis were total TB incidence and burden of MDR TB relative to total TB.

### Descriptive Analysis

We summarized participant characteristics with mean and SD for continuous variables and as counts and proportions for categorical variables. Participant home addresses were deidentified and converted to latitude and longitude coordinates by using the Google geocoding service and the tidygeocoder package in R (*25*). We obtained spatial polygons for the administrative units of Ho Chi Minh City from the Database of Global Administrative Areas (*26*). We mapped and aggregated individual TB cases and calculated average annual incidence of DS and MDR TB by ward.

### Spatial Autocorrelation

We assessed the presence, strength, and direction of spatial autocorrelation over the entire study area separately for DS and MDR TB incidence through the calculation of the global Moran I statistic. We assessed local spatial autocorrelation in these parameters through the calculation of the Getis-Ord Gi* statistic and Anselin Local Moran I. We used the Getis-Ord Gi* statistic to define spatial hot spots and cold spots relative to the null hypothesis of spatial randomness over the entire study area. In this analysis, we considered each ward in the context of its neighboring wards, forming a neighborhood. We compared the local sum of the values for the given parameter (e.g., DS TB incidence) for each of the wards in a neighborhood proportionally to the sum of the parameter values for all the wards in the study area. We designated neighborhoods with significantly higher parameter values than the entire study area as hot spots and neighborhoods with significantly lower parameter values than the entire study area as cold spots (*27*). The analysis using Anselin Local Moran I value further compared each ward to its neighborhood. We designated wards with high parameter values within neighborhoods with high values as high‒high clusters, wards with high values within neighborhoods with low values as high‒low outliers, wards with low values within neighborhoods with low values as low‒low clusters, and wards with low values within neighborhoods with high values as low‒high outliers (*28*). We applied false-discovery rate correction for multiple testing and spatial dependency to both local spatial autocorrelation analyses.

### Ecologic Analysis

We summarized continuous ward-level indicators with mean and SD or median and interquartile range, depending on skew. We summarized categorical indicators as counts and proportions. Exploratory analyses evaluated the relationship between ward-level demographic and socioeconomic indicators and total TB incidence and MDR TB case count relative to total TB case count. We assessed univariate associations between ward-level indicators and the natural logarithm of total TB incidence through the inspection of scatter plots and the calculation of the Spearman ρ for continuous indicators and by the Wilcoxon rank-sum test and analysis of variance for categorical indicators. We categorized continuous indicators with nonlinear associations with the outcome into tertiles. We included indicators associated with total TB incidence (p<0.05) in a multivariable negative binomial regression model for each outcome. We modeled ward-level TB incidence by including ward-level TB case count as the dependent variable with an offset term for ward population. We modeled ward-level MDR TB case count as a proportion of all TB cases by using MDR TB case count as the dependent variable with an offset term for total TB case count. Visualization of spatial autocorrelation in the residuals for each negative binomial regression model (measured by using the Moran I) demonstrated positive spatial autocorrelation in the residuals for both models, violating the assumption of independence. To account for that finding, we added a spatially autocorrelated random effects term to each model (using the centroid of each ward as latitude and longitude), assuming a Matérn covariance structure. We assessed additional assumptions, including the absence of multicollinearity and inequality in outcome means and variances. We compared model fit for the mixed-effects models and standard models using Akaike information criterion and scatter plots of the observed versus fitted values.

We conducted a sensitivity analysis to estimate the association between ward-level demographic and socioeconomic indicators and both outcomes using conditional autoregressive modeling. In contrast to the main analysis, in which spatial information was formatted as point data (i.e., latitude and longitude coordinates for the centroid of each ward), in the sensitivity analysis we reformatted spatial information as areal data, each ward represented by a spatial polygon surrounded by an administrative boundary. We defined ward neighbors by contiguity in administrative boundaries and converted neighborhood lists to an adjacency matrix by using binary weights to indicate the presence (1) or absence (0) of a neighbor. We incorporated the adjacency matrix into the negative binomial regression model as a random effects term to account for spatial autocorrelation between neighboring wards.

We conducted statistical analyses using R Studio (The R Foundation for Statistical Computing, https://www.r-project.org). We calculated statistics and conducted mapping by using ArcGIS Online (Environmental Systems Research Institute, https://www.esri.com).

## Results

### Descriptive Analysis

During January 1, 2020–April 30, 2023, a total of 36,089 persons registered for DS TB treatment and 1,451 persons for MDR TB treatment in Ho Chi Minh City. Of those, 49 participants with DS TB (0.1%) and 12 participants with MDR TB (0.8%) provided residential addresses outside Ho Chi Minh City and were excluded from the spatial analysis. Of the 37,540 total persons who registered treatment, 25,463 (67.7%) were male and 12,117 (32.3%) female; 30,268 (81%) were urban dwelling, and the mean (SD) age was 45 (16.5) years (Table 1). HIV co-infection was present in 5% of all participants (n = 1,692); this proportion was similar for both DS and MDR TB groups. Previous TB infection was reported by 4,721 (13%) of the participants given treatment for DS TB and 795 (55%) of the participants given treatment for MDR TB, although it is unknown how many previous infections were caused by drug-resistant TB.

**Table 1:** Characteristics of persons registered for TB treatment stratified by TB type, Ho Chi Minh City, Vietnam, January 1, 2020–April 30, 2023*

Characteristic	DS TB, n = 36,089	MDR TB, n = 1,451	Overall, n = 37,540
Age, y, mean (SD)	44.9 (16.6)	45.7 (14.1)	44.9 (16.5)
Sex, no. (%)			
F	11,732 (32.5)	385 (26.5)	12,117 (32.3)
M	24,357 (67.5)	1,066 (73.5)	25,423 (67.7)
HIV status, no. (%)			
Negative	27,745 (76.9)	1,333 (91.9)	29,078 (77.5)
Positive	1,609 (4.5)	83 (5.7)	1,692 (4.5)
Unknown	6,735 (18.7)	35 (2.4)	6,770 (18.0)
TB history, no. (%)			
No	31,344 (86.9)	655 (45.1)	31,999 (85.2)
Yes	4,721 (13.1)	795 (54.8)	5,516 (14.7)
Unknown	24 (0.1)	1 (0.1)	25 (0.1)
Residence type, no. (%)			
Urban	29,086 (80.6)	1,182 (81.5)	30,268 (80.6)
Rural	6,948 (19.3)	246 (17.0)	7,194 (19.2)

*DS TB, drug-susceptible tuberculosis; MDR TB, multidrug-resistant tuberculosis; TB, tuberculosis.

**Figure 1:**
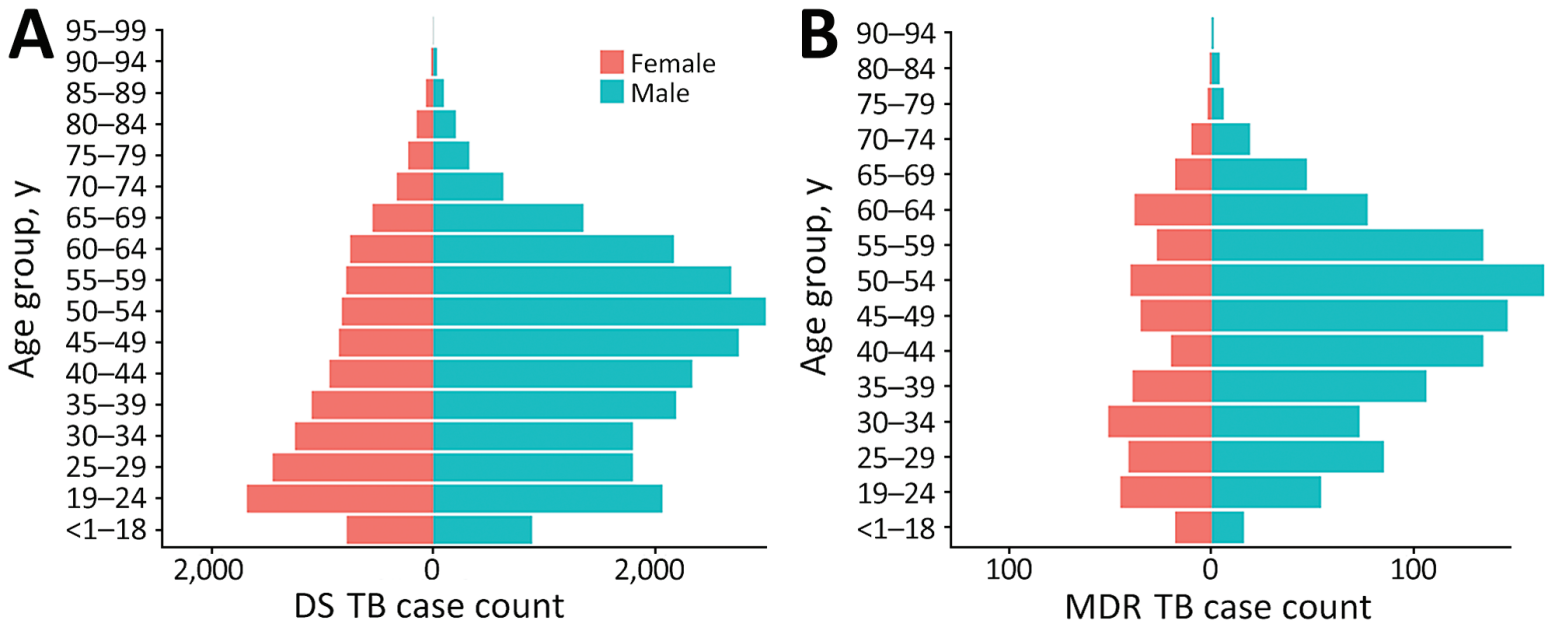
Population pyramids of age and sex distributions of participants registered for TB treatment in Ho Chi Minh City, Vietnam, January 1, 2020–April 30, 2023. A) DS TB; B) MDR TB. DS, drug-susceptible; MDR, multidrug-resistant; TB, tuberculosis.

Among 31,999 case-patients who had no history of TB, 640 (2%) registered for MDR TB treatment; 772 (14%) of the 5,516 case-patients who had a history of TB registered for MDR TB treatment. Asymmetric population pyramids demonstrated a greater DS and MDR TB burden among middle-aged to late middle–aged men, although the sex distributions were more symmetric in persons <40 years of age (Figure 1). The average annual incidence of notified DS TB in Ho Chi Minh City during this period was 121.4 (95% CI 119.1–123.7) cases/100,000 persons and of MDR TB was 4.8 (95% CI 4.4–5.4) cases/100,000 persons.

**Figure 2:**
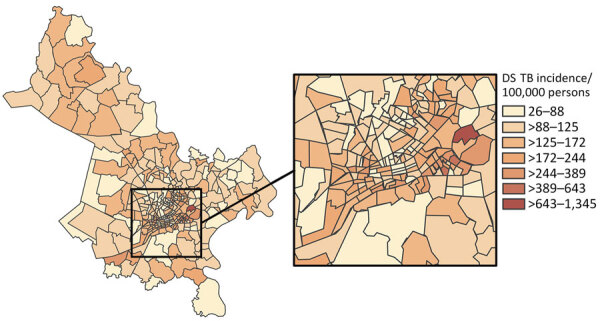
Choropleth map displaying geographic variation in average annual incidence (cases/100,000 persons) for DS TB, subdivided by ward, Ho Chi Minh City, Vietnam, January 1, 2020–April 30, 2023. Map does not include Cần Giờ district. Inset map shows location of study area in Vietnam. DS TB, drug-susceptible tuberculosis.

**Figure 3:**
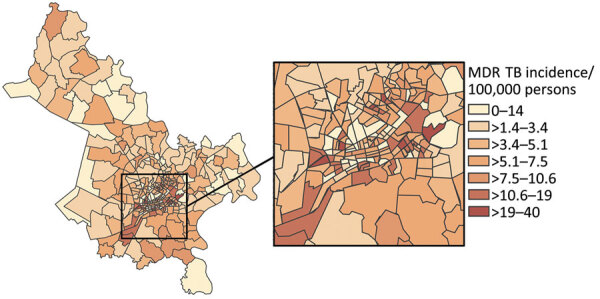
Choropleth map displaying geographic variation in average annual incidence (cases/100,000 persons) for MDR TB, subdivided by ward, Ho Chi Minh City, Vietnam, January 1, 2020–April 30, 2023. Map does not include Cần Giờ district. Inset map shows location of study area in Vietnam. MDR TB, multidrug-resistant tuberculosis.

We observed substantial spatial heterogeneity in DS and MDR TB average annual incidence across Ho Chi Minh City wards (Figures 2, 3). DS TB incidence (per 100,000 persons) ranged from 26.7 in Bình Lợi (District Bình Chánh) to 1,345.3 in An Khánh (District 2). Thirty-two wards recorded 0 MDR TB cases during the study period; ward 8 (District 11) showed MDR TB incidence of 31.7 cases/100,000 persons. In the overall study population, 3.9% (95% CI 3.7%–4.1%) of all TB cases were given treatment for MDR TB.

### Spatial Autocorrelation

**Figure 4:**
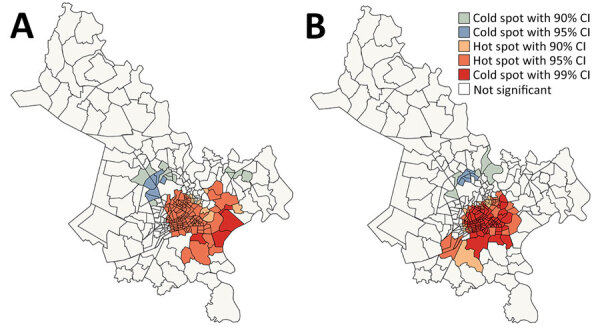
Spatial clustering of drug-susceptible (A) and multidrug-resistant (B) tuberculosis incidence, Ho Chi Minh City, Vietnam, January 1, 2020–April 30, 2023, based on the Getis-Ord GI* statistic.

**Figure 5:**
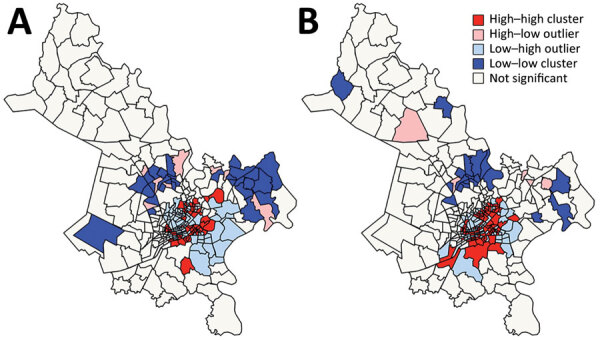
Spatial clusters and outliers of drug-susceptible (A) and multidrug-resistant (B) tuberculosis incidence, Ho Chi Minh City, Vietnam, January 1, 2020–April 30, 2023, based on the Anselin Local Moran I statistic.

The global Moran I statistic was 0.14 (p<0.001) for DS TB and MDR TB incidence, demonstrating weak positive global spatial autocorrelation for each parameter. This finding demonstrated that over the entire study area, wards with similar values for the above parameters (e.g., similar DS TB incidences) were located closer to each other than would be expected if the wards were randomly arranged (i.e., there was evidence of some spatial clustering for each parameter). However, the global Moran I provided no information about where these clustered wards were located or how the clustering of DS TB related to the clustering of MDR TB. We provide results of the hot spot analysis using the Getis-Ord Gi* statistic (Figure 4). Hot spots were evident in the central parts of Ho Chi Minh City for DS TB and MDR TB and cold spots to the north of the city center. Like the hot spots, the DS and MDR TB cold spots largely overlapped spatially. We provide Anselin Local Moran I to demonstrate wards in which TB incidence was congruent with the surrounding neighborhood (clusters) and wards in which TB incidence contrasted the surrounding neighborhood (outliers) (Figure 5). Heterogeneity in incidence, for DS TB and MDR TB, was evident even within hot spots and cold spots. For DS TB, most of the wards in the city center hot spot, when considered separately from their neighborhood, were low‒high outliers. A greater number of the wards that constituted the MDR TB hot spot were high‒high clusters, indicating more homogeneity within the MDR TB hot spots.

### Ward-Level Factors Associated with TB Burden

Wards in the highest tertile of TB incidence had the lowest male proportion of the population (47.6%), although the range of male proportion of the population between wards in the highest and lowest tertiles was small (47.6%–48.1%). Literacy rate (98.8%), proportion of homes that had a computer (65.2%), and lowest unemployment rate (2.8%) were also lowest in those wards (Table 2). Those wards had the highest proportion of the population 30–59 years of age (45.5%), population density (32,117 persons/km^2^), number of persons per household (3.6), and HIV prevalence (0.9%). Indicators strongly associated with TB incidence in the univariate analyses, and subsequently included in the final multivariable models, were male proportion of the population, proportion of the population 30–59 years of age, average number of persons per household, literacy rate, unemployment rate, and HIV prevalence (Appendix).

**Table 2:** Ward-level demographic and socioeconomic indicators stratified by tertiles of overall TB incidence, Ho Chi Minh City, Vietnam, January 1, 2020–April 30, 2023*

Indicator	Overall TB incidence			
	1st tertile, median incidence 84/100,000 persons, n = 105	2nd tertile, median incidence 120/100,000 persons, n = 105	3rd tertile, median incidence 187/100,000 persons, n = 105	Overall, n = 315
Male proportion of population, mean (SD)	48.1 (1.86)	48.2 (1.82)	47.6 (2.78)	48.0 (2.21)
Proportion of population 30–59 years old, mean (SD)	44.1 (2.23)	44.2 (2.02)	45.5 (1.52)	44.6 (2.04)
Residence type, no. (%)				
Urban	86 (81.9)	89 (84.8)	88 (83.8)	263 (83.5)
Rural	19 (18.1)	16 (15.2)	17 (16.2)	52 (16.5)
Location, no. (%)				
City center	23 (21.9)	22 (21)	24 (22.9)	69 (22)
Peripheral	82 (78.1)	83 (79)	81 (77.1)	246 (78)
Total population, median (IQR)	26,050 (12,402–40,289)	25,575 (13,354–42,067)	16,911 (11,190–25,068)	22,383 (12,397–36,880)
Population density, persons/km^2^, median (IQR)	27,537 (9,203–44,241)	20,810 (6,323–41,535)	32,117 (13,005–46,854)	27,781 (8,233–44,812)
Average no. persons per household, mean (SD)	3.51 (0.319)	3.55 (0.269)	3.62 (0.321)	3.56 (0.307)
Literacy rate, median (IQR)	99.3 (98.7–99.6)	99.3 (98.7–99.6)	98.8 (97.7–99.3)	99.2 (98.5–99.6)
Unemployment rate, mean (SD)	3.25 (1.17)	3.10 (1.05)	2.76 (0.747)	3.04 (1.02)
Proportion of homes that had a computer, median (IQR)	71.7 (55.4–77.6)	71.0 (59.3–76.5)	65.2 (59.3–73.2)	71.0 (59.3–76.3)
HIV prevalence, median (IQR)	0.49 (0.34–0.84)	0.48 (0.34–0.93)	0.93 (0.45–1.29)	0.49 (0.36–0.95)

*IQR, interquartile range; TB, tuberculosis.

In a multivariable negative binomial regression model with mixed effects, in contrast to the unadjusted association, the male proportion of the population was strongly associated with total TB incidence (incidence rate ratio 1.05, 95% CI 1.02–1.08), and each percentage increase in HIV prevalence was associated with a 77% increase in TB incidence (incidence rate ratio 1.77, 95% CI 1.54–2.03) (Table 3). None of the selected indicators were significantly associated with MDR TB case counts relative to total TB case counts. The mixed-effects models including spatially autocorrelated random effects terms demonstrated better fit than the standard models, and estimates from the sensitivity analysis were similar to those of the main analysis (Appendix).

**Table 3:** Adjusted incidence rate ratios for association between ward-level indicators and total TB incidence and MDR TB case count relative to total TB case count, Ho Chi Minh City, Vietnam, January 1, 2020–April 30, 2023*

Indicator	Total TB incidence (95% CI)	MDR TB case count (95% CI)†
Male proportion of population, %	1.05 (1.02–1.08)	0.99 (0.94–1.05)
Proportion of population 30–59 years old, %	1.02 (0.99–1.04)	1.03 (0.98–1.08)
Average no. persons per household	1.13 (0.98–1.31)	0.97 (0.76–1.25)
Literacy rate		
1st tertile	Referent	Referent
2nd tertile	1.06 (0.96–1.18)	1.05 (0.89–1.26)
3rd tertile	0.96 (0.86–1.07)	1.01 (0.83–1.25)
Unemployment rate, %	0.96 (0.92–1.00)	0.95 (0.87–1.03)
HIV prevalence, %	1.77 (1.54–2.03)	1.08 (0.85–1.38)

*MDR TB, multidrug-resistant tuberculosis; TB, tuberculosis. †Relative to total TB case count.

## Discussion

We characterized the burden of TB in Ho Chi Minh City with granular, ward-level descriptions of DS and MDR TB burden. Both DS and MDR TB were heterogeneously distributed throughout Ho Chi Minh City, forming geographic clusters of high incidence, predominantly concentrated in the city’s center. Total TB incidence at the ward level was strongly associated with HIV prevalence and more weakly associated with the proportion of the population that is male.

The asymmetric age and sex distributions among TB cases in Ho Chi Minh City we describe are consistent with the findings from the second Vietnam national TB prevalence survey, which confirmed prevalence of bacteriologically TB was 4 times greater in male than female patients and increased with age (*29*). Studies from Vietnam have also demonstrated a greater prevalence of latent TB in men than in women (*30*). However, the magnitude of this difference in prevalence by sex is smaller for latent TB than for active TB, emphasizing the role of sex differences in risk factors for disease progression. A recent substudy from the national TB prevalence survey specifically noted the stark differences in the prevalence of smoking (45% of men vs. 1% of women) (*31*) and drinking (44% of men vs. 1% of women) (*32*) in Vietnam as likely contributors to observed differences in the prevalence of active TB by sex (*33*). Sex differences, for both latent and active disease, remain incompletely understood but likely reflect the complex interplay between biologic, behavioral, and environmental factors (*34*). We demonstrated a 5% greater TB incidence per percentage increase in the proportion of the population that is male, suggesting sex-specific differences in risk might manifest at the population level.

We observed a 5% prevalence of TB and HIV co-infection, approximating previous regionally representative estimates (*14*,*35*). TB incidence was substantially greater with each percentage increase in HIV prevalence, emphasizing the potential contribution of HIV to the TB epidemic, even in settings with relatively low HIV prevalence.

Our incidence estimates for DS and MDR TB, derived from TB notifications, are markedly lower than the estimates of WHO for Vietnam (TB incidence 173 cases/100,000 persons, MDR TB 9.1 cases/100,000 persons) (*14*), despite evidence that Ho Chi Minh City has some of the highest TB incidences in the country (*15*,*16*). The WHO estimates are derived from multiple data sources, including prevalence surveys, case notification data, expert opinion about case detection gaps, and dynamic modeling (*36*). The differences between incidence estimates likely reflect a limitation of this study, the diagnostic gap—the difference between the true number of persons who became ill with TB and the number of persons who were registered for TB treatment (*1*). The diagnostic gap is a well-described barrier to TB control in Vietnam and has recently been exacerbated by COVID-19–related health system disruptions; <50% of predicted TB case-patients enrolled for treatment in 2021 (*1*).

TB incidence in Ho Chi Minh City was not associated with measures of poverty (literacy rates; unemployment rates; and proportion of homes that had a computer, a proxy for material wealth), even though poverty is a well-established risk factor (*37*). The central concentration of TB burden in Ho Chi Minh City was instead, in our data, related to factors such as sex distribution and HIV prevalence. This lack of association might reflect the poor representation of poverty and social deprivation by the variables included in our analysis (i.e., literacy rates are high across Ho Chi Minh City, even in poorer and rural areas [*23*]). It might also be that rapid equitable economic growth in Vietnam, coupled with a reduction in TB prevalence over the past 20 years, contributed to a reduction of the concentration of TB among poor households (*38*).

Our spatial analysis demonstrated substantial overlap in geographic clusters of DS and MDR TB incidence, raising interesting questions about the relationship between DS and MDR TB burden. Those findings might be consistent with the hypothesis that drug resistance largely emerges from DS TB de novo, and distributions of DS and MDR TB therefore related. Alternatively, the overlapping distributions might also be consistent with the hypothesis that most MDR TB is transmitted and that factors associated with the transmission of TB in general are geographically clustered. The lack of association between any demographic and socioeconomic indicators and MDR TB burden relative to total TB burden we describe potentially supports the latter hypothesis. Ultimately, it is likely that both de novo and transmitted resistance contribute to MDR TB burden. Enrichment of spatial data with genetic data will better demonstrate the relative contributions of each mechanism (*39*).

The first limitation of this study is that we used public sector registry data to identify TB cases and therefore excluded persons who had undiagnosed TB, potentially biasing our sample selection toward groups who are more likely to manifest signs or symptoms when symptomatic. Furthermore, we had no data on private-sector TB diagnoses, estimated to represent 8% of all TB cases in Ho Chi Minh City (*40*). Participants in our study were only geolocated through their home addresses. However, several studies have demonstrated the role of transmission outside the home with the emergence of genetic data demonstrating geographically unrelated, cryptic transmission networks mediated by mobility-linked locations in high-burden settings (*41*,*42*). Future work on the transmission of TB in Ho Chi Minh City will benefit from whole-genome sequencing–derived genetic data being generated by a parallel, related study. The degree to which our findings are relevant to other settings is uncertain, but it is likely that the dynamics in Ho Chi Minh City are not markedly different from other major cities with similar economic metrics in Southeast Asia, where nearly half the world’s TB patients reside (*1*).

In summary, we characterized the demographic profile of persons with DS and MDR TB in Ho Chi Minh City and mapped parts of the city most affected. Our findings provide a starting point for deeper research into TB acquisition and transmission dynamics and spatially informed TB control interventions in Ho Chi Minh City, Vietnam, and the greater southeastern region of Asia.

Appendix Additional information on spatial analysis of drug-susceptible and multidrug-resistant cases of tuberculosis, Ho Chi Minh City, Vietnam, 2020–2023.
