# Supplementary material for: Spatial Analysis of Drug-Susceptible and Multidrug-Resistant Cases of Tuberculosis, Ho Chi Minh City, Vietnam, 2020–2023
Source: Emerg Infect Dis. 2024 Mar;30(3):499–509. doi: 10.3201/eid3003.231309 (PMC10902525; doi:10.3201/eid3003.231309)
Supplement: Appendix — Additional information on spatial analysis of drug-susceptible and multidrug-resistant cases of tuberculosis, Ho Chi Minh City, Vietnam, 2020–2023. [file 23-1309-Techapp-s1.pdf]

Article DOI: https://doi.org/10.3201/eid3003.231309

EID cannot ensure accessibility for Supplemental Materials supplied by authors. Readers who have difficulty accessing supplementary content should contact the authors for assistance.

Spatial Analysis of Drug-Susceptible and Multidrug-Resistant Cases of Tuberculosis, Ho Chi Minh City, Vietnam, 2020–2023

Appendix

Methods

Definitions of Included Variables

For participants treated for DS-TB, where data was sourced from the NTP register, HIV status was defined as positive if participants self-reported their HIV status as positive or if the HIV test conducted at treatment initiation was positive. HIV status was defined as negative if participants reported no previous history of HIV infection and the HIV test conducted at treatment initiation was negative. History of previous TB was defined as 'yes' if participants self-reported any previous TB infection at any site before their current diagnosis and defined as 'no' if participants reported no previous TB infection. For participants treated for MDR-TB, sourced from the OUCRU cohort study database, HIV status was defined as positive if participants had a positive HIV test at enrollment into the study and negative if the HIV test was negative. Previous TB was defined as for participants treated for DS-TB.

For the ward-level analysis, the unemployment rate was defined as the proportion of persons older than 15 who were without work and were available for work. Population density was defined as the number of residents per square kilometer and literacy rate was defined as the proportion of persons above the age of 15 who could, with understanding, both read and write a short simple statement about their everyday life. Location was labeled as 'city center' for wards consisting the commercial, commuting and socializing hubs of Districts 1, 3, 4 and 10 and as 'peripheral' for all other wards. The socioeconomic indicators used as covariates in the ecologic analysis were principally selected based on the extent of information available from the Ho Chi

Minh City Statistics Office. Several markers of material wealth in addition to the proportion of homes that owned a computer were available (proportion of homes that owned a television, proportion of homes that owned a mobile phone and proportion of homes that owned a motorcycle). There was limited variation in the values of these indicators across districts however (all >90%) and we thus opted to select the proportion of homes that owned a computer (range 35% to 80%) as a single marker of material wealth.

Results

Ward-Level Factors Associated with TB Burden

The male proportion of the population, the proportion of the population 30 to 59 years of age, the average number of persons per household, the unemployment rate and HIV prevalence all demonstrated significant linear associations with the natural logarithm of total TB incidence (Appendix Figure 2). Population density, literacy rate, and the proportion of homes that owned a computer did not demonstrate linear relationships with the natural logarithm of total TB incidence and were subsequently categorized into tertiles. Among the categorical indicators, only literacy rate was associated with total TB incidence ($p = 0.01$) with p values for population density, the proportion of homes that owned a computer, residence type and location 0.42, 0.19, 0.35 and 0.31, respectively.

Model Fit

For the outcome total TB incidence, the inclusion of the spatially autocorrelated random effects term reduced the AIC to 3,140 from an AIC of 34,583 in the standard model. Fitted values in the mixed-effects model approximated the observed values more closely than in the standard model (Appendix Figure 3, panels A, B). A similar effect was demonstrated for the outcome MDR-TB case count relative to total TB case count where AIC decreased from 7,138 in the standard model to 1,362 in the mixed-effects model. Improvement in the proximity of fitted values to observed values was also notable for the mixed-effects model in comparison to the standard model, although the approximation of fitted values to observed values was poor – even for the mixed-effects model (Appendix Figure 3, panels C, D).

Sensitivity Analysis

In the sensitivity analysis, which used areal data and conditional autoregressive modeling to incorporate spatial autocorrelation as an adjacency matrix random-effects term, only HIV prevalence was strongly associated with total TB incidence (IRR 1.72, 95% CI 1.50–1.96). None of the selected demographic and socioeconomic indicators were associated with MDR-TB case counts relative to total TB case counts (Appendix Table).

Appendix Table. Adjusted incidence rate ratios and 95% CIs for the association between ward-level indicators and total TB incidence and MDR-TB case count relative to total TB case count, derived from sensitivity analysis*

Indicator	Total TB incidence, 95% CI	MDR-TB case count (relative to total TB case count), 95% CI
Proportion of population that is male (%)	1.02 (0.99–1.05)	0.97 (0.92–1.03)
Proportion of population 30–59 years of age (%)	1.02 (0.99–1.04)	1.04 (1.00–1.09)
Average number of persons per household	1.14 (0.98–1.32)	0.97 (0.74–1.26)
Literacy rate		
1st tertile	1 (reference)	1 (reference)
2nd tertile	1.07 (0.97–1.18)	1.07 (0.89–1.28)
3rd tertile	0.98 (0.88–1.10)	1.04 (0.84–1.28)
Unemployment rate (%)	0.96 (0.92–1.00)	1.02 (0.94–1.11)
HIV prevalence (%)	1.72 (1.50–1.96)	1.15 (0.90–1.47)

*MDR-TB, multidrug-resistant tuberculosis; TB, tuberculosis.

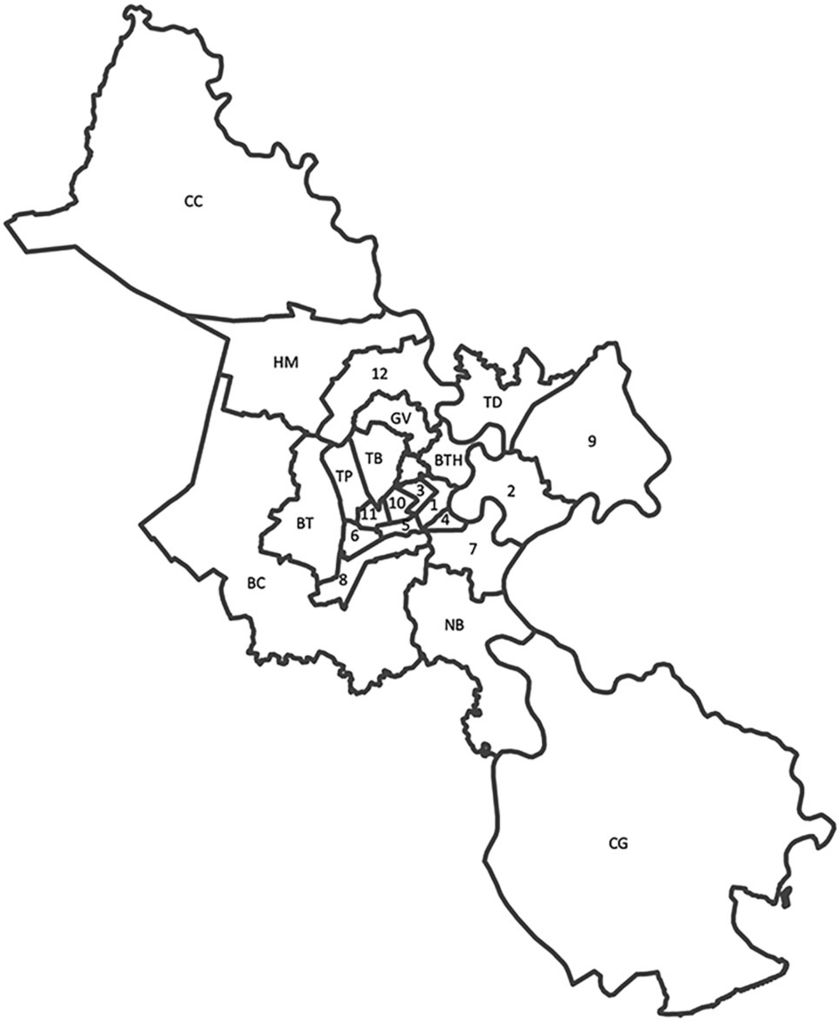

Appendix Figure 1. Ho Chi Minh City, Vietnam, administrative boundaries, subdivided by Districts 1–12. BT, Bình Tân; BTH, Bình Thạnh; GV, Gò Vấp; TB, Tân Bình; TD, Thủ Đức; TP, Tân Phú (all urban); BC, Bình Chánh; CC, Củ Chi; CG, Cần Giờ; HM , Hóc Môn; NB, Nhà Bè (all rural).

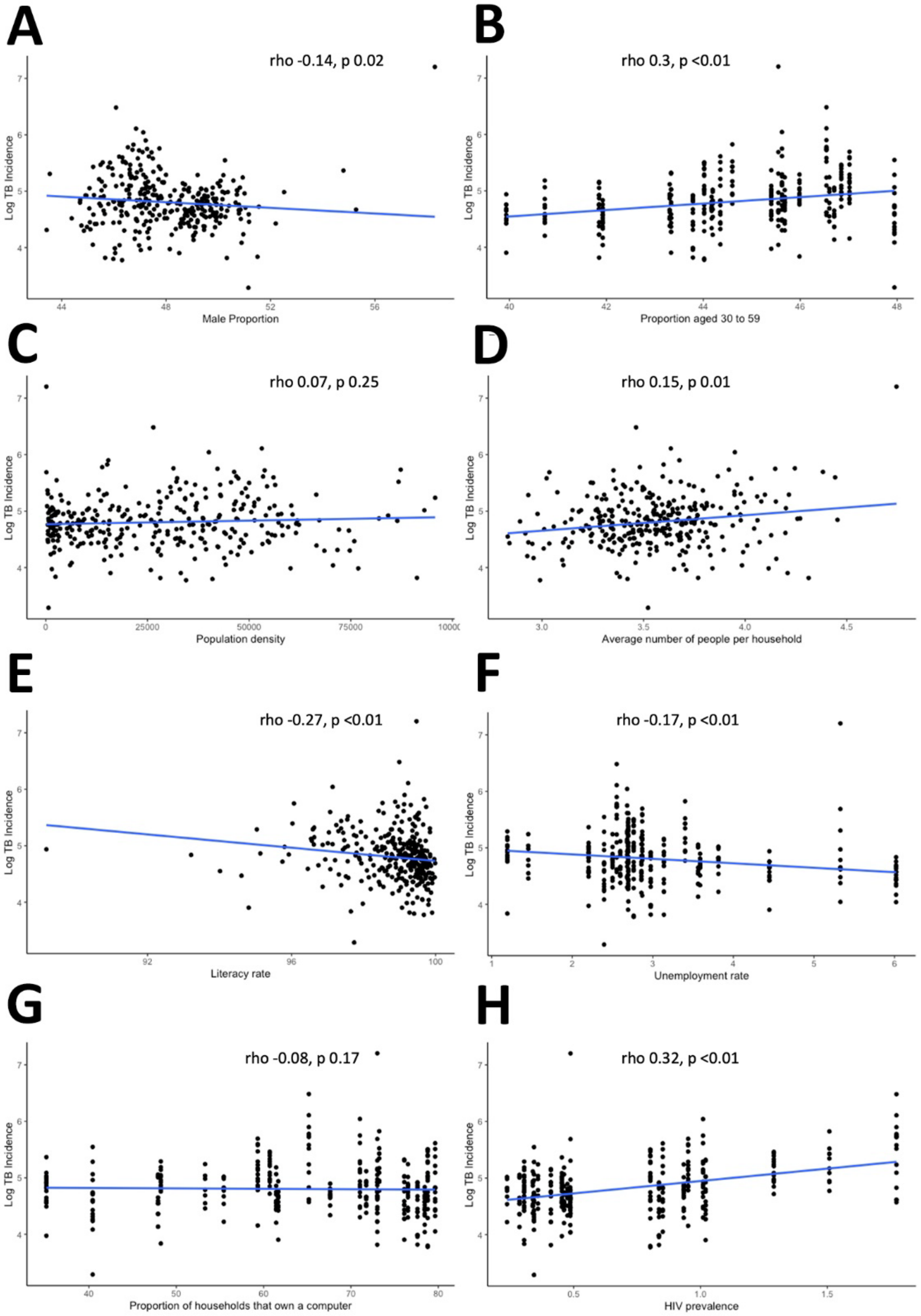

Appendix Figure 2. Scatter plots demonstrating univariate associations between ward-level demographic and socioeconomic indicators and natural logarithm of total TB incidence. Plots include robust line of best fit and Spearman correlation coefficient with corresponding p value. A, proportion of the population that is male; B, proportion of the population 30–59 years of age; C, average number of persons per household; D, unemployment rate; E, HIV prevalence; F, unemployment rate; G, proportions of households that own a computer; H, HIV prevalence.

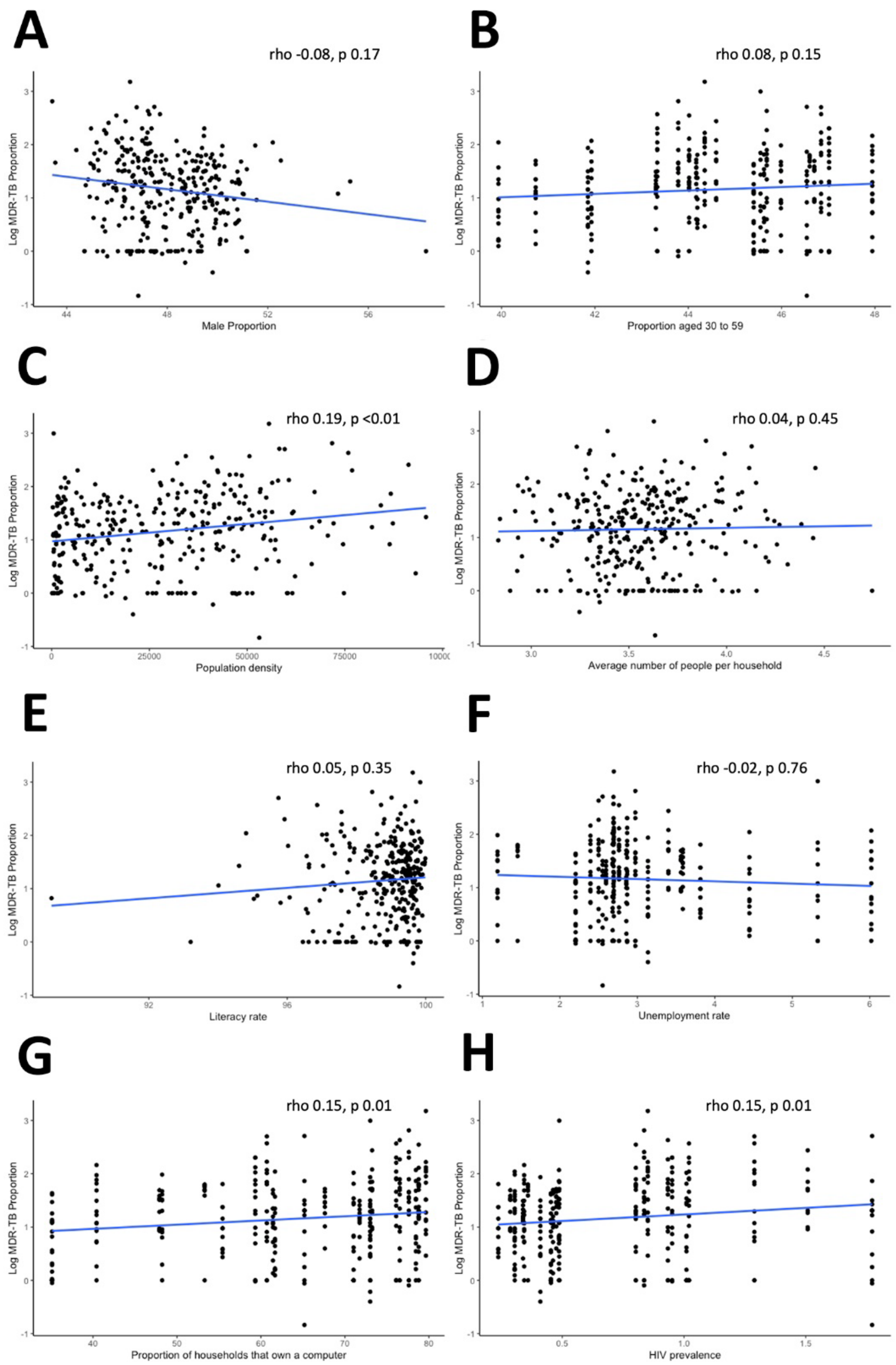

Appendix Figure 3. Scatter plots demonstrating univariate associations between ward-level demographic and socioeconomic indicators and the natural logarithm of proportion of all cases that are MDR-TB. Plots include robust line of best fit and Spearman correlation coefficient with corresponding p value. A, male proportion of the population; B, proportion of the population 30–59 years of age; C, population density; D, average number of persons per household; E, literacy rate; F, unemployment rate; G, proportions of households that own a computer; H, HIV prevalence.

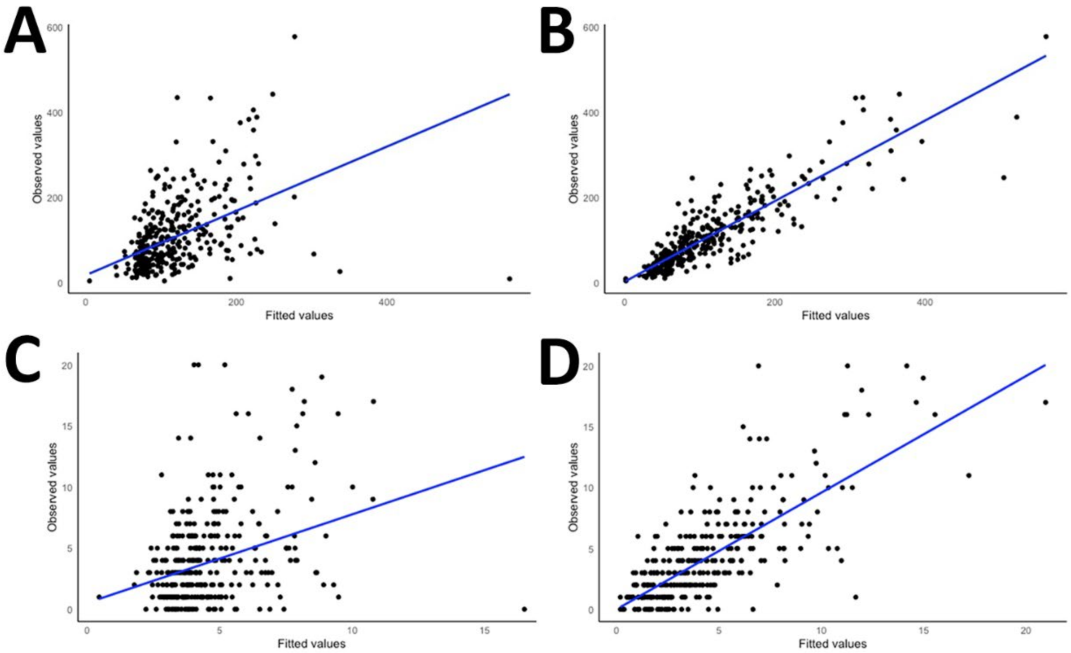

Appendix Figure 4. Scatter plots of fitted versus observed values for 4 negative binomial regression models. Plots include lines of best fit. A) Total TB incidence: standard model; B) Total TB incidence: mixed-effects model; C) MDR-TB versus total TB: standard model; D) MDR-TB versus total TB: mixed-effects model.
